# Environmental Signal-Dependent Regulation of Flowering Time in Rice

**DOI:** 10.3390/ijms21176155

**Published:** 2020-08-26

**Authors:** Jae Sung Shim, Geupil Jang

**Affiliations:** School of Biological Sciences and Technology, Chonnam National University, Gwangju 61186, Korea; jsshim@chonnam.ac.kr

**Keywords:** drought, environmental signals, flowering time, photoperiod, regional adaptation, rice, temperature

## Abstract

The transition from the vegetative to the reproductive stage of growth is a critical event in the lifecycle of a plant and is required for the plant’s reproductive success. Flowering time is tightly regulated by an internal time-keeping system and external light conditions, including photoperiod, light quality, and light quantity. Other environmental factors, such as drought and temperature, also participate in the regulation of flowering time. Thus, flexibility in flowering time in response to environmental factors is required for the successful adaptation of plants to the environment. In this review, we summarize our current understanding of the molecular mechanisms by which internal and environmental signals are integrated to regulate flowering time in *Arabidopsis thaliana* and rice (*Oryza sativa*).

## 1. Introduction

Flowering time is largely determined by environmental factors and is strongly associated with crop yield and quality [1,2,3,4]. Early or late flowering in rice causes huge reductions in grain production due to insufficient vegetative growth or poor fertility. To maximize reproductive success and grain production, flowering time must be precisely regulated by integrating internal and external cues. Among the various environmental cues, photoperiod, i.e., the duration of the light period, is the most reliable seasonal cue by which plants anticipate upcoming changes in environmental conditions. Therefore, the pathway used to sense and respond to photoperiod is a regulator of flowering time in plants [4,5]. In addition to the photoperiodic pathway, plants possess an autonomous pathway to induce flowering in the absence of external cues. The factors involved in the autonomous pathway regulate expression of the *FLOWERING LOCUS C* (*FLC*) floral repressor through RNA processing, transcriptional and epigenetic regulations [6,7].

During the domestication of rice, the area of rice cultivation expanded from tropical and subtropical regions of Asia to temperate regions at higher latitudes [8]. The adaptation of rice to these latitudes was enabled by the acquisition of photoperiod-insensitive traits [9,10,11]. Several environmental stresses also participate in the regulation of flowering time by affecting the expression of genes associated with the photoperiodic flowering pathway [3,12,13]. Thus, identifying key elements controlling photoperiod sensitivity and investigating their responses to other environmental stresses will build a foundation for breeding elite rice varieties with the attributes needed to adapt to particular environments.

In this review, we describe the molecular mechanisms underlying the regulation of photoperiodic flowering time in the model plant *Arabidopsis thaliana* and the important crop rice. We then discuss how naturally occurring variations have contributed to regional adaptation in rice. Finally, we discuss how drought and temperature signals are integrated into these flowering time pathways, and suggest that these pathways could be fine-tuned in efforts to breed rice varieties with improved drought and heat tolerance.

## 2. Regulation of Flowering Time in Arabidopsis

**Figure 1:**
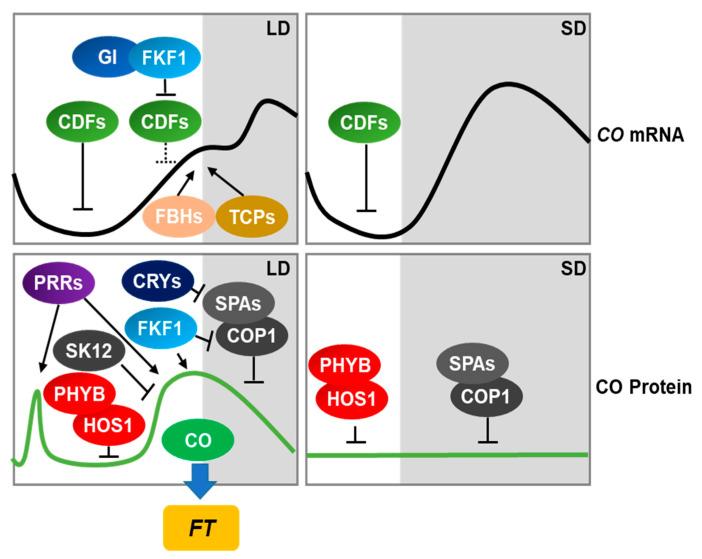
Photoperiodic regulation of *FT* expression is mediated by *CO* in Arabidopsis. Photoperiodic flowering in Arabidopsis is primarily mediated by transcriptional and posttranslational regulation of the transcription factor gene *CONSTANS* (*CO*). Under inductive long-day conditions, the CYCLING DOF FACTOR (CDF)-dependent repression of *CO* expression is diminished by the blue light-dependent FKF1-GI complex. FBHs and TCPs then induce *CO* expression. CO protein stability is further regulated by PHYB, SK12, PRRs, FKF1, CRYs, COP1, and SPAs. PHYB mediates red light dependent CO destabilization. SK12 interacts with CO and phosphorylate it for destabilization. During the night, CO is degraded by the COP1-SPAs complex. This COP1-SPA-dependent CO degradation is inhibited by FKF1 and COP1 in the presence of blue light. In addition, the circadian clock component PRRs interact with and stabilize CO. Once CO has been stabilized in the afternoon, it activates the transcription of *FT*, which in turn induces flowering. Under non-inductive short-day conditions, CDFs continuously repress the transcription of *CO* during the day. In addition, the PHYB-HOS1 and COP1-SPAs complexes inhibit CO accumulation, thereby preventing flowering under short-day conditions.

The mechanisms controlling flowering time have been extensively investigated in Arabidopsis, a model dicot plant [5,12]. Flowering time largely depends on changes in the expression of the *FLOWERING LOCUS T* (*FT*) gene [14,15] (Figure 1). *FT* encodes a mobile signaling molecule, also known as florigen, that promotes the transition from the vegetative to the reproductive stage of growth [16,17]. *FT* is synthesized in the leaves and moves to the shoot apex to induce flowering. In Arabidopsis, *FT* expression is induced under long-day conditions, which in turn accelerates flowering, whereas short-day conditions restrict *FT* expression to very low levels to attenuate floral initiation [14]. This day-length-dependent expression of *FT* represents a major molecular mechanism that precisely determines flowering time.

The day-length-dependent regulation of *FT* expression is mainly controlled by the zinc finger-type transcriptional activator CONSTANS (CO) [18,19,20]. To restrict *FT* expression to the afternoon under long days, both the transcriptional and posttranslational regulation of *CO* are important. *CO* transcripts accumulate from the afternoon into the night. The temporal expression of *CO* is achieved by the action of CYCLING DOF FACTORs (CDFs) [21,22,23]. These transcriptional repressors of flowering directly repress *CO* transcription in the morning. The Arabidopsis *cdf1 cdf2 cdf3 cdf5* quadruple mutant shows accelerated flowering under both short- and long-day conditions and elevated *CO* expression in the morning regardless of photoperiod [21], indicating that the precise control of *CDF* expression is crucial for regulating *CO* expression and flowering time. CDF1 recruits the TOPLESS co-repressor to the promoter of *CO* through its N-terminal domain to repress *CO* transcription [24].

*CDF1* expression is controlled by multiple circadian clock components [25,26,27]. In the morning, *CDF1* expression is induced by CIRCADIAN CLOCK ASSOCIATED1 (CCA1) and LATE ELONGATED HYPOCOTYL (LHY). PSEUDO-RESPONSE REGULATORs (PRRs) then repress *CDF1* transcription in the afternoon. These regulatory mechanisms determine the diurnal oscillation patterns of *CDF1* expression. In addition to transcriptional regulation, posttranslational regulation of CDFs is also involved in the day-length-dependent regulation of *CO* transcription. CDF-dependent repression of *CO* transcription is released by the circadian clock-controlled proteins FLAVIN-BINDING, KELCH REPEAT, F-BOX1 (FKF1), and GIGANTEA (GI) [23]. FKF1 and GI form a complex in a blue light-dependent manner. Both FKF1 and GI accumulate to high levels in the afternoon under long-day conditions, leading to the formation of the day-length-specific FKF1-GI complex. The FKF1-GI complex mediates the ubiquitin-dependent degradation of CDF1 responsible for the repression of *CO* transcription. Once CDF proteins have been degraded by the FKF1-GI complex, FLOWERING BHLHs (FBHs) and TEOSINTE BRANCHED/CYCLOIDEA/PROLIFERATING CELL NUCLEAR ANTIGEN FACTORs (TCPs) induce *CO* transcription [28,29,30].

The posttranslational regulation of CO is another key mechanism used by plants to sense external environmental signals to induce *FT* transcription. External light signals are integrated into the flowering time pathway through multiple photoreceptors. PHYTOCHROME B (PHYB) is involved in the red light-dependent destabilization of CO [20]. PHYB forms a complex with HIGH EXPRESSION OF OSMOTICALLY RESPONSIVE GENES 1 (HOS1), a RING-finger E3 ubiquitin ligase that degrades CO in the morning [31,32]. In contrast to red light, far-red and blue light stabilize CO. The far-red light-dependent stabilization of CO is mediated by PHYA [20]. Cryptochrome 2 (CRY2) senses blue light and stabilizes CO [33]. In the presence of blue light, CRY2 forms a complex with CONSTITUTIVE PHOTOMORPHOGENIC 1 (COP1) and SUPPRESSOR OF PHYA-105 1 (SPA1) to inhibit COP1-SPA1-dependent CO degradation [33]. During the night, the COP1-SPA complex actively degrades CO to prevent flowering under short-day conditions [34].

The COP1-dependent degradation of CO is associated with the phosphorylation of this protein [35,36]. Both phosphorylated and unphosphorylated forms of CO are present in Arabidopsis plants throughout the day. The levels of phosphorylated CO are lower during the night than in the daytime, and phosphorylated CO accumulates in *cop1* mutants, suggesting that phosphorylation destabilizes CO via COP1 activity [35]. The recent identification of SHAGGY-like kinase 12 (SK12) highlighted the importance of CO phosphorylation in flowering time regulation. SK12 was identified as a CO-interacting partner by coimmunoprecipitation-coupled liquid chromatography-tandem mass spectrometry. SK12 phosphorylates CO at Thr^119^ to promote its degradation.

In addition to these regulatory mechanisms, time-specific regulatory mechanisms also affect CO stability. CO is stabilized only in the early morning and late afternoon under long-day conditions, a process involving FKF1 and PRRs. FKF1 directly interacts with CO through its LOV domain; this interaction is enhanced by blue light [19]. Thus, both the circadian clock-controlled accumulation of FKF1 and the light period determine the degree of FKF1-dependent CO stabilization. *PRR*s, encoding master regulators of circadian oscillators, are expressed at specific times of day [37]. Beginning at dawn, *PRR9*, *PRR7*, *PRR5*, and *TOC1* are sequentially expressed through transcriptional feedback loops. Like FKF1, PRRs interact with and stabilize CO [38]. The accumulation of CO in the early morning and late afternoon is completely abolished in the *toc1 prr5 prr7 prr9* quadruple mutant. In the *toc1 prr5 prr7* triple mutant, CO accumulates in the early morning but not in the afternoon, indicating that PRR9 is responsible for stabilizing CO in the early morning. The mutation of all four *PRR*s alleviates the blue light- and far-red light-dependent stabilization of CO, which is mediated by FKF1, CRY2, and PHYA [38]. The *cop1* mutation is epistatic to *prr* mutations with respect to CO accumulation, suggesting that PRRs might also suppress COP-mediated degradation of CO during the day [38]. FKF1 physically interacts with COP1 and inhibits its activity by interfering with its dimerization [39]. The molecular mechanism by which PRRs inhibit COP1-dependent CO degradation remains to be elucidated.

## 3. Regulation of Flowering Time in Rice

**Figure 2:**
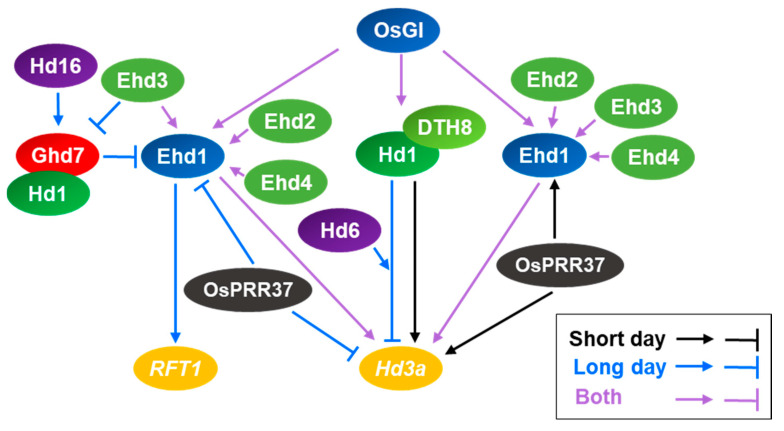
The regulatory network controlling *Hd3a* and *RFT1* expression in rice. Flowering is regulated by two distinct pathways in rice, *Hd1-Hd3a* and *Ghd7-Ehd1-Hd3a/RFT1*. Under short-day conditions, Hd1 positively regulates *Hd3a* expression. DTH8 interacts with Hd1 to help upregulate *Hd3a* expression. The expression of *Ehd1*, which encodes another activator of *Hd3a*, is induced by Ehd2, Ehd3, and Ehd4 regardless of photoperiod. Under long-day conditions, Hd1 is converted to a negative regulator of *Hd3a* expression. Hd6 enhances the negative function of Hd1 on the *Hd3a* expression under long day conditions. In addition, Ghd7 acts as a repressor of *Ehd1* expression, leading to the suppression of *RFT1* expression. The Ghd7-dependent suppression of *Ehd1* expression is enhanced by *Hd16*. Hd1 physically interacts with Ghd7 to repress *Ehd1* expression under long-day conditions. OsPRR37 affects flowering by suppressing the transcription of *Ehd1* and *Hd3a* under long day conditions, but activating expression of *Ehd1* and *Hd3a* under short day conditions.

Several quantitative trait loci (QTLs) for heading date have been mapped in rice in an effort to explore the molecular mechanisms underlying the floral transition in this crop. These studies have uncovered several key regulators involved in regulating flowering time (Figure 2). *Heading date 3a* (*Hd3a*) and *RICE FLOWERING LOCUS T1* (*RFT1*), orthologs of Arabidopsis *FT*, encode rice florigens. Similar to Arabidopsis FT, the products of these genes are produced in leaves and move to the shoot apical meristem to induce flowering in rice [40,41,42]. In rice, *Hd3a* expression promotes flowering under short-day conditions, whereas *RFT1* expression is required for flowering under long-day conditions [40,41]. Two major pathways control the expression of *Hd3a* and *RFT1*: the *Hd1-Hd3a* pathway, which resembles the Arabidopsis *CO-FT* module, and the rice-specific *Ghd7*-*Ehd1-Hd3a-RFT1* pathway.

*Heading date 1* (*Hd1*), identified by map-based cloning, was the first flowering time gene to be reported in rice [43]. *Hd1* encodes a zinc finger protein and is an ortholog of Arabidopsis *CO*. Like Arabidopsis *CO*, *Hd1* is crucial for the day-length-specific induction of flowering in rice. Hd1 is a bi-functional protein that either represses or activates the expression of the rice florigen gene *Hd3a.* The Hd1-dependent regulation of *Hd3a* expression is controlled by the circadian clock and light signaling. Under inductive short-day conditions, *Hd1* is mainly expressed at night, whereas under non-inductive long-day conditions, *Hd1* is highly expressed from night to dawn [44]. The diurnal expression of *Hd1* is regulated by OsGI, an ortholog of Arabidopsis GI [44]. Hd1 upregulates *Hd3a* expression in the dark. Hd1 is converted from an activator to a repressor of *Hd3a* expression in the presence of light, a process mediated by phytochrome [45]. In the phytochrome-deficient mutant *photoperiod sensitivity 5* (*se5*), Hd1 positively regulates *Hd3a* expression regardless of day length [46]. Thus, diurnal expression patterns governed by the circadian clock and the light-dependent functional conversion of Hd1 facilitate the induction of *Hd3a* expression under short-day conditions.

The repressive activity of Hd1 is enhanced by the kinase activity of Hd6, but it appears that Hd6 indirectly affects the repressive activity of Hd1 on *Hd3a* expression under long-day conditions [47]. Rice possesses homologs of Arabidopsis FKF1, GI, and CDFs, which regulate the day-length-dependent expression of *CO* in Arabidopsis. Even though OsFKF1 interacts with OsGI and OsDOF12, OsFKF1 is involved in the transcriptional regulation of rice-specific flowering genes rather than *Hd1* [48]. The different consequences of the OsFKF1-dependent regulation of the flowering pathway in rice vs. Arabidopsis may be due to OsDOF12. OsDOF12 functions in photoperiodic flowering by regulating the transcription of *Hd3a* but not *Hd1* [49].

The posttranslational regulation of Hd1 in rice is also different from that of Arabidopsis CO. In Arabidopsis, CO is actively degraded by the COP1-SPA complex in the dark to inhibit *FT* expression [34]. Similarly, Hd1 protein level is also low during night in rice [50]. However, Hd1 protein was detected at similar levels in both day and night in *Hd1* overexpressing plants, indicating that Hd1 protein seems to be stable in the dark in rice [51]. Therefore, COP1 and SPA may have different functions in rice. Peter Pan Syndrome (PPS) is a rice ortholog of Arabidopsis COP1. Like COP1, PPS1 is also involved in the transition from the vegetative to the reproductive phase, but PPS1-dependent regulation is independent of the *Hd1-Hd3a/RFT1* pathway [52]. In addition to PPS1, Heading date Associated Factor 1 (HAF1), a C3HC4 RING domain-containing E3 ligase, controls Hd1 protein stability in rice [50]. HAF1 physically interacts with Hd1, and high levels of Hd1 were detected in *haf1* mutants. Thus, HAF1 might mediate the ubiquitination of Hd1 for 26S proteasome-dependent degradation.

Flowering in rice is also regulated by a distinct molecular pathway consisting of the rice-specific B-type response regulator Early heading date 1 (Ehd1) and Ghd7 (Grain number, plant height, and heading date) [53,54]. Ehd1 promotes flowering by upregulating *Hd3a* and *RFT1* expression under both short-day and long-day conditions. Several upstream regulators control *Ehd1* expression in rice. For example, Ehd2, Ehd3, and Ehd4 positively regulate *Ehd1* expression under both short-day and long-day conditions [55,56,57]. By contrast, Ghd7 negatively regulates *Ehd1* expression [54]. The expression of *Ghd7* gradually increases in response to increasing day length [58]. This day-length-dependent accumulation of *Ghd7* transcripts is mediated by phytochrome [59].

LH8/DTH8/Hd5/Ghd8 is a CCAAT-box-binding transcription factor that suppresses flowering in rice under long-day conditions by downregulating *Hd3a* expression [60,61]. Interestingly, DTH8 upregulates *Hd3a* expression under short-day conditions [61]. They also found that the genetic effect of *DTH8* on regulating rice flowering time is dependent on its genetic background. The bi-functionality of DTH8 could be explained by its physical interaction with Hd1 [62]. The DTH8-Hd1 complex increases H3K27 trimethylation at the *Hd3a* locus and represses *Hd3a* expression. Thus, DTH8-Hd1 complex formation is important for the long-day-specific transcriptional repression of *Hd3a* by Hd1. DTH8 also indirectly suppresses the expression of *Ehd1*, encoding an upstream regulator of *Hd3a* [60,61]. A recent study proposed that DTH8 binds to the promoter of *Ghd7* to induce its expression [63]. This binding is enhanced in the presence of Hd1. Therefore, the Hd1-DTH8 complex might suppress the expression of *Hd3a* through both direct repression and indirect repression via *Ghd7*.

## 4. Natural Variations that Help Rice Adapt to Different Latitudes

Photoperiodic sensitivity is an agronomically important trait that adjusts the timing of the reproductive transition in response to local climate conditions. Several genetic studies have been performed to identify key variations responsible for changes in photoperiodic sensitivity in rice. Genetic analysis identified several polymorphisms on the promoter and coding region of *Hd3a* from a core rice collection [64]. Among the seven types of promoters, four groups sharing several polymorphisms allow higher expression of *Hd3a*. In addition, six types of *Hd3a* coding sequences have been identified, but none cause amino acid changes in the functional domain of Hd3a. These findings suggest that *Hd3a* is highly conserved among rice cultivars, but its upstream regulatory mechanisms cause variations in the photoperiodic sensitivity of different rice cultivars. Unlike *Hd3a*, a natural variation in *RFT1* abolishes its interaction with 14-3-3 proteins, which are intercellular receptors of rice florigens [65,66]. The presence of different *RFT1* variants is closely associated with the regional distribution of different rice cultivars. Most *japonica* and *indica* cultivars grown at higher latitudes contain functional *RFT1*, whereas nonfunctional *RFT1* is present in some *indica* cultivars grown at lower latitudes [65].

*Ehd1,* a upstream activator of *Hd3a* and *RFT1*, contains a highly conserved coding sequence present in all rice cultivars examined except the Chinese rice cultivar DANYU [64]. An amino acid substitution (G219R) in the GARP domain of Ehd1 decreases its DNA binding activity [53]. A higher degree of polymorphism has been identified in the *Hd1* locus than in *Hd3a* and *Ehd1* [9,43,64,67]. Rice cultivars harboring functional *Hd1* alleles exhibit elevated *Hd3a* expression and earlier flowering, whereas those carrying nonfunctional *Hd1* alleles show lower *Hd3a* expression and later flowering [64]. Phylogenetic analysis revealed that the nonfunctional *Hd1* allele was introduced into rice during the expansion of rice cultivation in tropical regions [9]. Indeed, the nonfunctional allele of *Hd1* facilitates the proper regulation of rice flowering time in tropical regions [67].

Mutations in *Ghd7*, *OsPRR37*, and *Hd16* also accelerated the diversification of flowering time within subspecies [68,69,70]. *Ghd7* is a key element involved in the adaptation of rice cultivars to higher latitudes. Ghd7 downregulates the expression of *Ehd1*, *Hd3a*, and *RFT1* under long-day conditions to delay flowering [54,71]. Two nonfunctional alleles of *Ghd7* were identified in rice cultivars grown in central China [9,54,72]. Nonfunctional *DTH7/OsPRR37* alleles were identified in many European and Asian rice cultivars [69]. OsPRR37 acts as a negative regulator of the transcription of *Ehd1* and *Hd3a* under long day conditions, but acts as a positive regulator of the expression of *Ehd1* and *Hd3a* under short day conditions [73]. Rice cultivars harboring nonfunctional *DTH7*/*OsPRR37* alleles exhibit early flowering due to the absence of DTH7/OsPRR37-dependent repression of *Ehd1* and *Hd3a* expression [68,69]. Some rice cultivars grown at higher latitudes in Asia have nonfunctional *Ghd7* and *DTH7/OsPRR37* alleles [69]. Eliminating these two major repressors causes extremely early flowering under long-day conditions, making it possible to cultivate rice plants even in the northernmost regions. A naturally occurring allele of *Hd16* decreases its phosphorylation activity. The mutation causes lower phosphorylation of Ghd7, leading to elevated expression of *Ehd1*, *RFT1*, and *Hd3a* and early flowering under long-day conditions [74]. These observations suggest that the combination of different alleles of flowering genes facilitated the rapid expansion of rice plants to broader regions.

## 5. Environmental Stresses and Flowering

### 5.1. Impact of Drought on Flowering

Plants have evolved the ability to endure drought stress using an array of physiological, morphological, and biochemical adaptations in processes such as drought escape, drought avoidance, and drought tolerance [75]. Drought escape is the ability of a plant to complete its life cycle before drought becomes severe. Plasticity of flowering time in response to drought has been reported in several plant species [76,77]. As in other plants, mild drought conditions accelerate flowering in rice [13], including both *indica* (Zhenshan 97 and Minghui 63) and *japonica* (Zhonghua 11) rice cultivars. Drought-induced flowering is achieved by the abscisic acid (ABA)-dependent upregulation of two florigen genes, *Hd3a* and *RFT1*. ABA-deficient *phd3-1* mutants and plants with suppressed *PHYTOENE DESATURASE* (*PDS)* expression are insensitive to drought-induced flowering. The ABA-dependent induction of *Hd3a* and *RFT1* transcription is mediated by the ABA-inducible bZIP transcription factor OsbZIP23 [13]. Overexpressing *OsbZIP23* resulted in the upregulation of *Ehd1* and the downregulation of *Ghd7*, indicating that drought-induced flowering is mainly achieved through *OsbZIP23*-mediated transcriptional regulation.

Several other bZIP transcription factors are also involved in drought-mediated flowering time regulation in rice, including OsFD1/OsbZIP77 and OsbZIP72 [78,79,80]. OsFD1/OsbZIP77 promotes flowering by forming a complex with Hd3a and RFT1 in the shoot apical meristem [81]. In addition to developmental cues, *OsFD1/OsbZIP77* expression is also induced by ABA treatment [78]. Furthermore, OsFD1/OsbZIP77 interacts with the SnRK2 protein SAPK10. SnRK2 proteins transmit ABA signals to a downstream transcriptional network by phosphorylating ABA-dependent transcription factors [82]. OsFD1/OsbZIP77 is a phosphorylation target of SAPK10 *in vitro*. Further studies are needed to determine whether SAPK10 also phosphorylates OsFD1/OsbZIP77 in vivo and whether this phosphorylation alters the activity of OsFD1/OsbZIP77 [78]. Similar to OsFD1/OsbZIP77, OsbZIP72, a positive regulator of ABA responses and drought tolerance in rice [79], formed a complex with Hd3a, RFT1, and GF14c when heterologously expressed in yeast [83]. However, the contribution of these proteins to flowering time regulation under drought stress is not clearly understood.

Various circadian clock components are also involved in drought-mediated flowering time regulation. TIMING OF CAP EXPRESSION 1 (TOC1) and GI are involved in drought responses in plants [77,84,85]. In Arabidopsis, TOC1 binds to the promoter of the ABA-related gene *ABAR* and regulates its oscillation patterns. ABA treatment during subjective daytime strongly induced *TOC1* expression, whereas ABA treatment during subjective night had no clear effect on *TOC1* expression, indicating that ABA responses, at least *TOC1* expression, are gated by the circadian clock [84]. The induction of *TOC1* expression could be mediated by the MYB96 transcription factor [86]. MYB96 directly binds to the *TOC1* promoter to activate *TOC1* expression. This circadian gating of ABA responses governed by *TOC1* is important for plant adaptation to drought. Like Arabidopsis *TOC1*, *OsTOC1* transcription is induced by both ABA and drought treatment in rice [13]. Transgenic rice plants overexpressing *OsTOC1* showed earlier flowering than control plants in response to both drought and ABA treatment. The accelerated flowering in *OsTOC1*-overexpressing plants could be explained by the upregulated expression of *Hd3a* and *RFT1* [13]. Drought also induces the expression of *OsGI* and *OsERF3,* which encode positive regulators of flowering, but suppresses the expression of *OsPRR37*, which acts as a negative regulator of flowering in rice [13].

Unlike the results described above, another study indicated that exposure to drought stress reduces *Hd3a*, *RFT1*, and *Ehd1* expression and delays flowering in rice [87]. This discrepancy could have been due to the different experimental conditions used in these studies, such as different degrees of drought stress and different growth conditions. For example, Du et al. detected no significant decrease in relative water content in the leaves of rice plants grown under their drought conditions [13], whereas Galbiati et al. used more severe drought conditions [87]. In addition, the drought-induced gene *OsABF1* (*ABA RESPONSIVE ELEMENT BINDING FACTOR 1*) encodes a repressor of flowering that suppresses *Ehd1* expression by directly activating *OsWRKY104* [80]. These findings suggest that plants use different survival strategies depending on the severity of drought stress. Further investigations of the integration of drought signals into the flowering pathway in rice are needed to breed new rice varieties with increased adaptation to unpredictable variations in the environment.

### 5.2. Impact of Temperature on Flowering

Temperature changes act as strong signals that regulate flowering time by modulating the expression of multiple flowering genes in plants. In general, high ambient temperatures accelerate flowering, whereas low ambient temperatures delay flowering [88,89]. Thus, a precise understanding of the thermosensory pathway controlling flowering time at the molecular level is crucial for improving the regional fitness of rice plants. Nonetheless, little is known about the temperature-dependent regulation of flowering in rice.

Temperature signals are integrated into multiple points in the flowering pathway. In Arabidopsis, high ambient temperatures upregulate *FT* expression and induce flowering under both inductive long-day and non-inductive short-day conditions [90,91,92]. The histone H2A variant H2A.Z plays important roles in the temperature-dependent regulation of *FT* expression [90,91,92]. The occupation of the *FT* promoter by H2A.Z nucleosomes is controlled by ambient temperature. H2A.Z nucleosomes associate with the *FT* promoter at low temperature, which in turn represses *FT* expression. This repression is relieved and *FT* is highly expressed at high temperature [92]. The transcription factor PHYTOCHROME INTERACTING PROTEIN 4 (PIF4) is involved in upregulating *FT* expression at high temperature [92,93]: high temperature promotes the association of PIF4 with the *FT* promoter, thereby increasing expression of *FT*. The evening complex, comprising ELF3-ELF4-LUX ARRHYTHMO (LUX), plays a crucial role in regulating PIF4 activity. ELF3 physically interacts with PIF4 and attenuates the binding of PIF4 to its target promoters [94]. In addition, the evening complex directly represses *PIF4* transcription [95]. High temperature decreases the binding of the evening complex to the *PIF4* promoter to induce its expression [96,97].

Arabidopsis SVP (SHORT VEGETATIVE PHASE) also determines the temperature-dependent expression of *FT* [98,99]. SVP is a MADS-domain transcription factor that inhibits flowering in Arabidopsis. SVP interacts with the MADS-box gene *FLOWERING LOCUS M* (*FLM*), which acts as an inhibitor of flowering [100]. In Arabidopsis, *FLM* is transcribed into two splicing variants: *FLM-β* and *FLM-δ*. Low temperature increases the *FLM-β/FLM-δ* ratio, allowing the majority of SVP to form a complex with FLM-β, leading to the inhibition of *FT* expression. By contrast, high temperature decreases the FLM-β/FLM-δ ratio. The SVP-FLM-δ complex shows impaired DNA binding. Thus, SVP-dependent suppression of *FT* is relieved at higher temperatures [100,101].

Daily temperatures dynamically change under natural conditions. In tropical and subtropical environments, the ambient temperature gradually increases from the morning through late afternoon and decreases thereafter. Temperature changes occur at different times of day depending on the season and light conditions. Daily temperature changes might contribute to flowering time regulation. This concept was proposed based on discrepancies in flowering time between plants grown under natural field conditions vs. laboratory conditions [102]. To test the potential effects of daily temperature fluctuations on flowering time regulation, the expression of flowering time genes was investigated under temperature conditions that mimic natural temperature fluctuations [103,104,105,106]. At constant lower temperatures, *FT* transcript levels were reduced and plants flowered later compared to normal temperature conditions [98]. Intermittent drops in temperature during the day also lead to the repression of *FT* expression and delayed flowering [107]. In addition, cold night-time temperatures under long-day conditions lead to the repression of *FT* expression and delayed flowering [104], primarily due to the increased production of the SVP-FLM-β complex, as described above.

Interestingly, cooler night-time temperatures increase *CO* transcript levels. The upregulation of *CO* is achieved by the action of FBH transcription factors, as the induction of *CO* in response to cold temperature treatment at night is diminished in *fbh* quadruple mutants [104]. Song et al. (2018) more precisely determined the effect of temperature fluctuations on flowering time regulation [105]. The authors used temperature ramping (a gradual increase in temperature from dawn to afternoon, followed by a decrease) to mimic temperature fluctuations that occur under natural conditions. Like constant cold temperatures at night, daily temperature fluctuations led to the repression of *FT* expression and delayed flowering [105].

As in Arabidopsis, flowering in rice is also regulated by ambient temperature. When rice plants are grown at constant temperature, they start to flower later under low vs. higher temperature conditions [89,108]. The transcription of the key floral activator genes *Ehd1*, *Hd3a*, and *RFT1* is reduced at low temperature, whereas the transcription of *Ghd7*, a floral repressor gene, is induced at low temperature. However, *Hd1* expression is not significantly affected by changing temperatures. These observations suggest that temperature signals are integrated into the flowering pathway through Ehd1 and Ghd7, two major transcriptional regulators, rather than Hd1. Recently, Guo et al. reported that four key flowering genes (*Hd1*, *OsPRR37*, *DTH8*, *Hd6*) are involved in flowering time plasticity on temperature [109]. The effect of these four genes on the regulation of flowering time is dependent on the temperature. For example, Koshihikari allele of *Hd1* acts as a negative regulator in most of the environments, and the negative effect is stronger at lower temperature. On the other hand, *OsPRR37* allele from Koshihikari promotes flowering under high temperature, but delays flowering under lower temperature. These suggest that multiple flowering genes are involved in temperature-dependent regulation of flowering time in rice, which facilitates the adaptation of rice to the regions with different temperature ranges. Further studies are needed to elucidate the molecular pathway underlying temperature-dependent flowering time regulation in rice.

## 6. Conclusions and Future Perspectives

In this review, we discussed recent advances in the field of flowering time regulation in Arabidopsis and rice. As in Arabidopsis, flowering time in rice is determined based on sensed changes in photoperiod. Rice is a facultative short-day plant. Thus, flowering in rice is accelerated under short-day conditions through the *Hd1-Hd3a* pathway. In addition, rice possesses the *Ghd7-Ehd1-Hd3a/RFT1* pathway, which allows flowering to be induced under various photoperiods. In the past two decades, most genes with major effects on flowering time have been identified through genetic approaches. These studies revealed various natural variations in flowering time genes in rice. Depending on the combination of these natural variations, rice plants exhibit different responses to the environment, making rice capable of withstanding a range of ecological and climatic conditions and one of the most widely cultivated crops in the world. This nucleotide sequence-level information could be used for the precision breeding of rice using new and emerging plant breeding technologies such as genome editing.

Nonetheless, further studies are needed to obtain a comprehensive understanding of the mechanisms regulating flowering time in rice. Exploring genetic information via genome-wide analysis, QTL mapping, and mutant analysis will continue to provide exciting insights into the genetic architecture of flowering time in rice. In addition, systematic evaluations of flowering time and other agronomic traits in various rice cultivars and natural variants will be important for precisely predicting flowering time in this crop [110], as well as for identifying new QTLs. Finally, molecular approaches combined with biochemical and biophysical techniques will identify novel components involved in regulating flowering time. This information will lay the foundation for designing strategies that enable plants to adapt to various environments, which in turn could improve grain production in the face of the extreme conditions caused by global climate change.
